# Ethylene is Involved in Symptom Development and Ribosomal Stress of Tomato Plants upon Citrus Exocortis Viroid Infection

**DOI:** 10.3390/plants9050582

**Published:** 2020-05-02

**Authors:** Francisco Vázquez Prol, M. Pilar López-Gresa, Ismael Rodrigo, José María Bellés, Purificación Lisón

**Affiliations:** Instituto de Biología Molecular y Celular de Plantas, Universitat Politècnica de València-Consejo Superior de Investigaciones Científicas, 46011 Valencia, Spain; fravazpr@posgrado.upv.es (F.V.P.); mplopez@ceqa.upv.es (M.P.L.-G.); irodrig@ibmcp.upv.es (I.R.); jmbelles@btc.upv.es (J.M.B.)

**Keywords:** CEVd, ethylene, ribosome, stress, viroids, biogenesis, rRNA, tomato, plants

## Abstract

Citrus exocortis viroid (CEVd) is known to cause different symptoms in citrus trees, and its mechanism of infection has been studied in tomato as an experimental host, producing ribosomal stress on these plants. Some of the symptoms caused by CEVd in tomato plants resemble those produced by the phytohormone ethylene. The present study is focused on elucidating the relationship between CEVd infection and ethylene on disease development. To this purpose, the ethylene insensitive *Never ripe* (*Nr*) tomato mutants were infected with CEVd, and several aspects such as susceptibility to infection, defensive response, ethylene biosynthesis and ribosomal stress were studied. Phenotypic characterization revealed higher susceptibility to CEVd in these mutants, which correlated with higher expression levels of both defense and ethylene biosynthesis genes, as well as the ribosomal stress marker *SlNAC082*. In addition, Northern blotting revealed compromised ribosome biogenesis in all CEVd infected plants, particularly in *Nr* mutants. Our results indicate a higher ethylene biosynthesis in *Nr* mutants and suggest an important role of this phytohormone in disease development and ribosomal stress caused by viroid infection.

## 1. Introduction

Viroids are the smallest known plant pathogens and consist of circular, highly structured, non-coding RNA molecules with autonomous replication that parasite the transcriptional machinery of their hosts [1,2]. Viroids can be classified into two families, the *Avsunviroidae*, whose members replicate in the chloroplasts and have a hammerhead-like structure, and the *Pospiviroidae*, characterized by a rod-like structure and replicating in the nucleus [3]. Citrus exocortis viroid (CEVd), which belongs to the *Pospiviroidae* family, consists of around 370 nucleotides and has a broad range of hosts including tomato plants (*Solanum lycopersicum* L.). CEVd symptoms in tomato include stunting, epinasty, midvein necrosis, chlorosis and leaf rugosity [4,5,6]. Some of these symptoms have been associated with those produced by ethylene, since plants exogenously treated with the ethylene–releasing compound ethephon, as well as mutants that over produce ethylene, display similar symptoms to those caused by CEVd [7,8,9]. However, the relationship between ethylene production and signaling, and the disease caused by CEVd, has not been deeply studied.

Ethylene (ET) is a small phytohormone involved in plant growth, development and stress response. Despite its low basal levels, a quick induction of its biosynthesis can be observed under biotic or abiotic stress and senescence [10]. Due to its gaseous nature, ethylene can easily move in the plant without transporters. Thus, biosynthesis appears to be the key step in the regulation of ethylene signaling. The ethylene biosynthetic pathway has two limiting steps: (1) the conversion of S-adenosylmethionine (SAM) to 1-aminocyclopropane carboxylic acid (ACC), catalyzed by ACC synthase (ACS), and (2) the oxidation of ACC to ethylene via ACC oxidase (ACO) [11,12]. There are several ACS and ACO encoding genes in tomato plants. Among these, *ACS2* and *ACS6* as well as *ACO1* are induced under biotic stress [7,13]. Early studies reported an increase in ethylene production upon an infection by CEVd in leaves and cell cultures of tomato as a result of ACS induction [14,15,16,17], suggesting a possible role of ethylene in CEVd disease development. In ethylene signaling, ethylene receptors constitutively block downstream response. These receptors become inactivated upon ethylene binding, allowing the activation of the ethylene response cascade [18]. Six ethylene receptors have been described to date in tomato, LeETR1-6, including receptor LeETR3, named Never ripe (NR). Tomato lines with a mutation in this receptor present an impaired ethylene perception, due to a single amino acid change in the ethylene-binding domain (Pro36Leu) [19,20,21]. These mutants, named *Never ripe* (*Nr*), have been used to study the relationship between ethylene signaling and disease development in different pathogens [22,23,24,25].

Viroid pathogenicity is a complex phenomenon and little is known about the molecular mechanisms leading to disease upon viroid infection. The relationship between viroids and ethylene as a source of either resistance or pathogenesis has only been superficially explored. Recent studies showed a slightly increased resistance to tomato chlorotic dwarf viroid (TCDVd) in *Nr* mutants [22], and pointed to an induction of ethylene-related genes upon potato spindle tuber viroid (PSTVd) infection [26]. Several works have also suggested a relationship between viroid infection and changes in the translation machinery, despite their lack of coding capability. Different viroids have been shown to interact with ribosomal proteins or elongation factors [27,28]. In particular, CEVd has been shown to produce alterations in the accumulation of the ribosomal proteins S3, S5 and L10, as well as in the translation of elongation factors eEF1A, eEF2 and eIF5A [29].

Our laboratory has recently uncovered evidence on the effect of CEVd on tomato ribosomal stress [30]. This type of stress is normally caused by anomalies in plant ribosome biogenesis that are associated with serious developmental alterations. In *Arabidopsis thaliana*, ribosomal stress is mediated by the NAC transcription factor ANAC082, which acts downstream of the perturbation of the biogenesis of the ribosome and leads to growth defects and developmental alterations [31,32]. Induction of *SlNAC082,* a tomato ortholog of *ANAC082*, was described in CEVd infected plants, pointing to the viroid as the first described pathogen causing ribosomal stress [30]. Eukaryotic ribosomes are formed by two subunits, the small subunit 40S, which consists of the 18S rRNA and approximately 33 ribosomal proteins, and the large subunit 60S, consisting of the 25S, 5.8S, 5S and approximately 47 ribosomal proteins [33,34]. During CEVd infection, we have described a defect in the processing of the 18S rRNA, impairing the assembly of the 40S subunit and thus altering ribosome biogenesis [30].

Ethylene involvement in translation is still unexplored. Some early studies in fruits point to an increase in protein translation after ethylene treatment by an increase in polyribosome size and ribosome synthesis [35,36,37]. Moreover, some studies have demonstrated the role of ethylene in the regulation of translational machinery by stopping the translation of certain genes [38]. The objective of the present study is to contribute to this knowledge by exploring the role of ethylene in the tomato defensive response against CEVd, and its involvement in ribosomal stress by using the ethylene insensitive mutants *Never ripe*.

## 2. Results

### 2.1. Never Ripe Tomato Mutants Are Hyper-Susceptible to CEVd Infection

**Figure 1:**
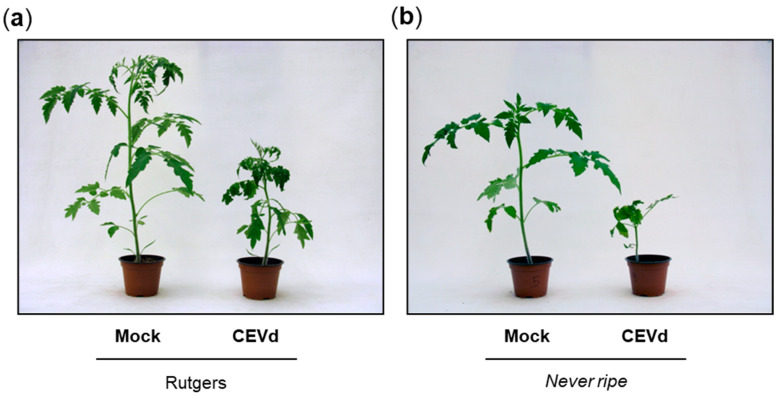
Symptomatology in Rutgers parental and *Never ripe* tomato plants at 20 days after citrus exocortis viroid (CEVd) inoculation. Representative phenotype observed in (**a**) wild type Rutgers plants and (**b**) *Never ripe* mutants.

To study the role of ethylene in the development of viroid symptoms, parental Rutgers and *Never ripe* tomato plants were infected with CEVd, and visually inspected for symptom development throughout the experiment. The typical symptomatology of tomato plants infected by CEVd consists of epinasty, stunting, leaf rugosity, midvein necrosis and chlorosis [5]. As Figure 1 shows, differences in symptom severity were observed between genotypes at 20 days post inoculation (dpi), with *Nr* mutants displaying a much more severe epinasty, stunting and leaf rugosity, and therefore appearing to be more susceptible to the viroid infection.

**Figure 2:**
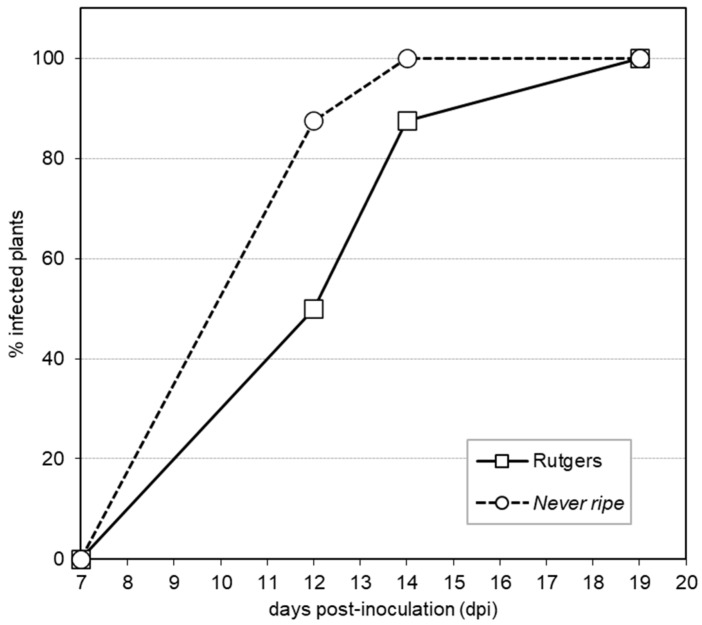
Disease development in Rutgers and *Never ripe* (*Nr*) plants infected with CEVd. Evolution of the percentage of tomato plants showing symptoms at the indicated days post inoculation (dpi). Data displayed correspond to one representative experiment.

To better quantify the observed *Nr* hyper-susceptibility, the percentage of plants displaying mild epinasty was tracked to obtain a graphic representation of disease development (Figure 2). The accelerated appearance of symptoms was observed in *Nr* mutants with 87.5% plants showing symptoms at 12 days post inoculation (dpi), when only 50% parental plants exhibited them. At 14 dpi, all the *Nr* mutant plants displayed symptoms, while 12.5% Rutgers plants remained symptomless. These results suggest an accelerated symptom appearance in *Nr* mutant tomato plants, confirming the higher susceptibility observed.

**Figure 3:**
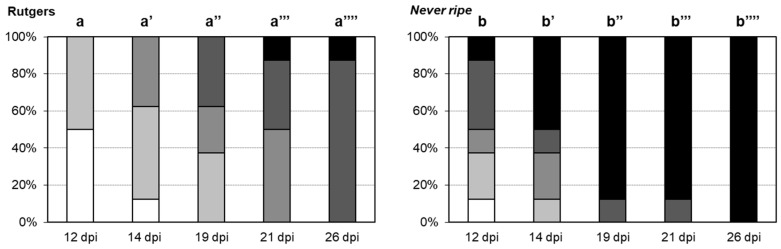
Disease severity of CEVd infected Rutgers and *Never ripe* tomato plants. Symptomatology was scored at 12, 14, 19, 21 and 26 days post inoculation (dpi), using the following scale: no symptoms (white), mild epinasty (light grey), severe epinasty and stunting (grey), leaf rugosity (dark grey), midvein necrosis and chlorosis (black). Data correspond to one representative experiment. Data were analyzed using a Mann–Whitney test and different letters indicate significant differences (*p* < 0.05). Number of (**’**) indicates different days.

To verify the statistical significance of the differences in the disease development shown in Figure 2, a scale of the disease severity was developed, scoring symptoms from mild (mild epinasty) to very severe (midvein necrosis and chlorosis), at different time points (see Materials and Methods). At the beginning of the viroid infection (12 dpi), 12.5% of the *Nr* mutants already showed very severe symptoms, whilst Rutgers plants remained symptomless or displayed mild symptoms (Figure 3). These differences continued throughout the experiment, with most *Nr* plants (87.5%) showing very severe symptoms by 19 dpi. Severe symptomatology was not observed in Rutgers plants until 21 dpi, and by the end of the experiment, all of the *Nr* plants, but only 12.5% of Rutgers plants showed severe symptoms. In conclusion, differences in symptom severity were statistically significant at any time between *Nr* and Rutgers tomato plants, correlating with symptom appearance, and thus confirming the hyper-susceptibility of *Nr* tomato mutants to CEVd infection.

**Figure 4:**
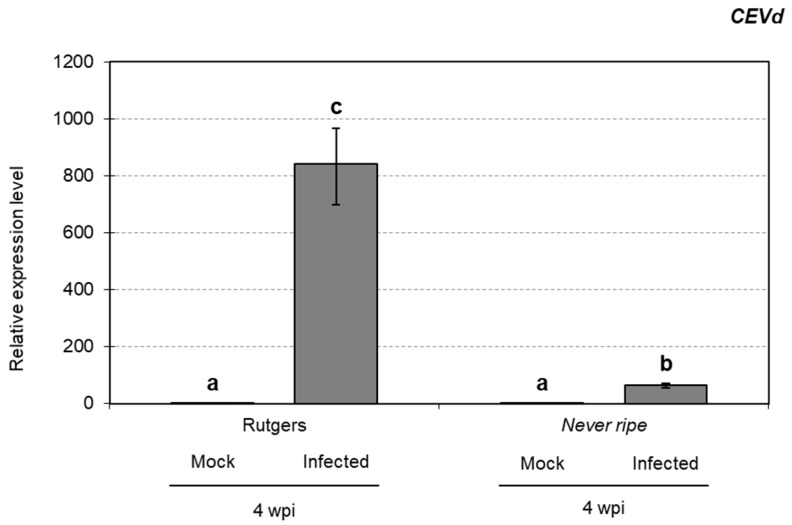
*CEVd* accumulation in Rutgers and *Nr* plants in CEVd-infected and mock plants determined by qRT-PCR four weeks post inoculation (wpi). Expression levels are relative to Rutgers mock plants and normalized to the tomato actin gene (accession AB199316). Data correspond to the mean of two or more independent plants ± SD of at least 3 technical replicates. Data displayed correspond to one representative experiment. Data were analyzed using a Mann–Whitney test and different letters indicate significant differences (*p* < 0.05).

To study the possible relationship between the observed symptomatology and pathogen accumulation, CEVd levels were analyzed in Rutgers and *Nr* infected tomato plants at 4 wpi by qRT-PCR (see Materials and Methods). Surprisingly, a higher accumulation of CEVd was observed in infected parental Rutgers plants at 4 wpi (Figure 4), when compared to the hyper-susceptible *Nr* mutants. These data indicate that symptom development appears not to be correlated to the accumulation of CEVd.

### 2.2. PR1 and ACS2 Are Highly Induced in Infected Nr Tomato Plants

To find out if *Nr* mutants, which have blocked ethylene perception, were also impaired in the activation of the defensive response against CEVd, the accumulation and expression of pathogenesis related protein 1 (*PR1;* accession X71592) [39], which has been described as a classical marker of plant defense that is rapidly induced in CEVd-infected tomato plants [40,41]. Besides, the induction of genes involved in ethylene biosynthesis was also analyzed.

**Figure 5:**
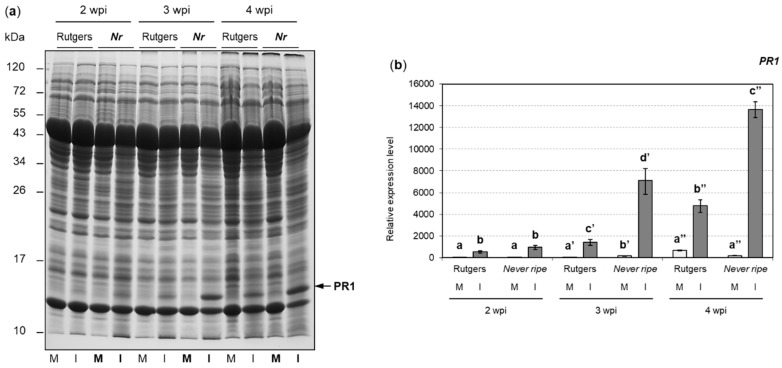
Pathogenesis related protein 1 (PR1) analysis at 2, 3, and 4 weeks post inoculation (wpi) in mock (M) and CEVd infected (I) Rutgers and *Never ripe* tomato leaves. (**a**) SDS-PAGE analysis of soluble proteins. Protein size markers (kDa) are indicated on the left. The arrow on the bottom right indicates the PR1 protein. (**b**) mRNA expression of *PR1* determined by qRT-PCR Expression levels are relative to Rutgers mock plants and normalized to the tomato actin gene. Results correspond to the mean of at least 2 independent plants ± SD of at least three technical replicates, and one representative experiment is displayed. Data were analyzed using a Mann–Whitney test and different letters indicate significant differences (*p* < 0.05). Number of (**’**) indicates different weeks.

For the analysis of defense proteins, both PR1 accumulation and *PR1* gene expression were measured in control and CEVd-infected tomato plants of both genotypes (see Materials and Methods), and data were statistically analyzed (Figure 5). As expected, accumulation of PR1 protein increased over time in all plants as the disease progressed, its high levels being even detectable by Coomassie Blue stain (Figure 5a). Interestingly, a higher accumulation of PR1 was observed for all time points in infected *Nr* mutants when compared to their parental Rutgers plants. Expression levels of *PR1* gene correlated with PR1 protein levels and the hyper-susceptibility observed in *Nr* mutants (Figure 5b). In fact, the induction of *PR1* was statistically higher in the infected *Nr* mutants at 3 and 4 wpi than in the wild type Rutgers.

**Figure 6:**
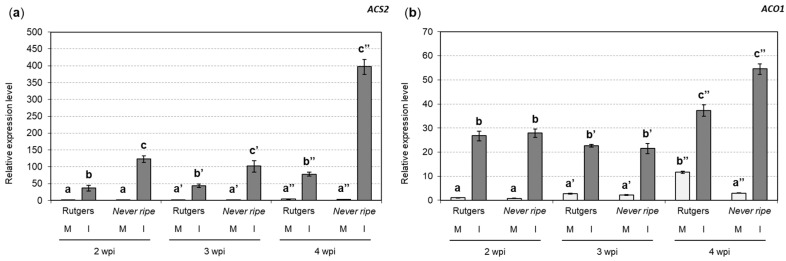
mRNA expression of ethylene biosynthesis enzymes in Rutgers and *Nr* plants from mock (M) and CEVd infected (I) plants determined by qRT-PCR, at 2, 3, and 4 weeks post inoculation (wpi). Relative expression levels of both (**a**) *ACS2* and (**b**) *ACO1* genes. Expression levels are relative to Rutgers mock plants and normalized to the tomato actin gene. Data correspond to the mean of at least two independent plants ± SD of at least three technical replicates. Results from one representative experiment are shown. Data were analyzed using a Mann–Whitney test and different letters indicate significant differences (*p* < 0.05). Number of (**’**) indicates different weeks.

The contribution of ethylene in Rutgers tomato plants after CEVd infection has already been described [5,15]. However, less is known about the role of ethylene in CEVd-infected *Nr* mutants. To analyze the ethylene implication in the development of CEVd symptoms in tomato plants, the expression levels of *1-aminocyclopropane-1-carboxylate synthase 2* (*ACS2*; accession X59145.1) (Figure 6a) and *1-aminocyclopropane-1-carboxylate oxidase* (*ACO1*; accession X58273.1) (Figure 6b) genes were analyzed, as the induction of these isoforms has been described upon pathogen attack, and at the onset of the climacteric stage in tomato fruits, where a high accumulation of ethylene is produced [7,42,43]. The expression levels of *ACS2* (Figure 6a) were higher in CEVd-infected plants when compared with the corresponding non-infected plants, at any time point and for both genotypes. More interestingly, these levels were always statistically higher in infected *Nr* mutants when compared to the corresponding infected Rutgers plants. Similarly, *ACO1* expression levels (Figure 6b) were also higher in CEVd infected plants when compared to the non-infected control plants, but showed no significant differences between infected *Nr* mutants and Rutgers plants.

Our results indicate a distinctive increase in both PR1 accumulation and ethylene biosynthesis gene expression during CEVd infection, and this induction is enhanced in *Nr* mutants.

### 2.3. Ethylene Production Is Increased in Nr Tomato Mutants upon CEVd Infection

**Figure 7:**
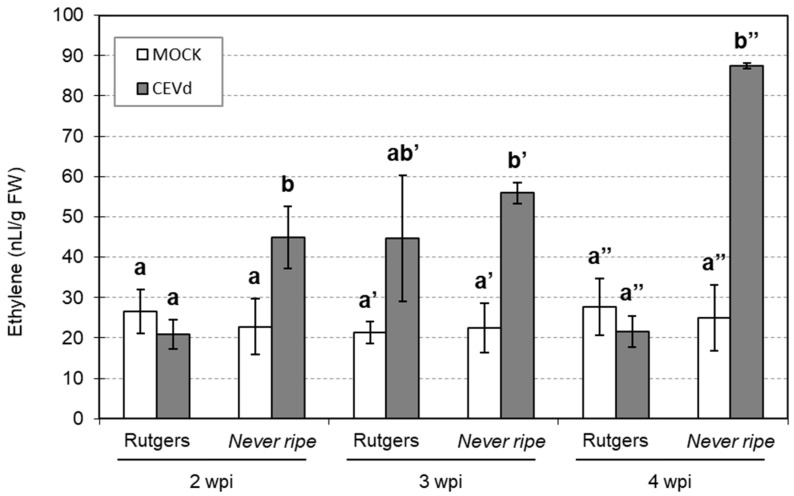
Ethylene emission levels in both mock and CEVd infected Rutgers and *Nr* tomato leaves at two, three, and four weeks post inoculation (wpi). Data correspond to the mean ± SD of at least two biological replicates. Results are shown from one representative experiment. Data were analyzed using a Mann–Whitney test and different letters indicate significant differences (*p* < 0.05). Number of (**’**) indicates different weeks.

Ethylene levels were measured to correlate the observed increase in the expression of ethylene biosynthesis genes in *Nr* infected plants with the emitted ethylene (Figure 7). In Rutgers tomato plants, no significant differences in ethylene levels were observed between control and CEVd infected plants at any time. In contrast, *Nr* infected mutants showed higher ethylene levels at any time point, which correlates with the enhanced expression levels of *ACS2* (Figure 6a).

Our results indicate that ethylene levels do not correlate with *ACS2* and *ACO1* gene expression in Rutgers plants, but closely correlate in *Nr* mutants.

### 2.4. Ribosomal Stress Is Enhanced in Never Ripe Tomato Mutants upon CEVd Infection

**Figure 8:**
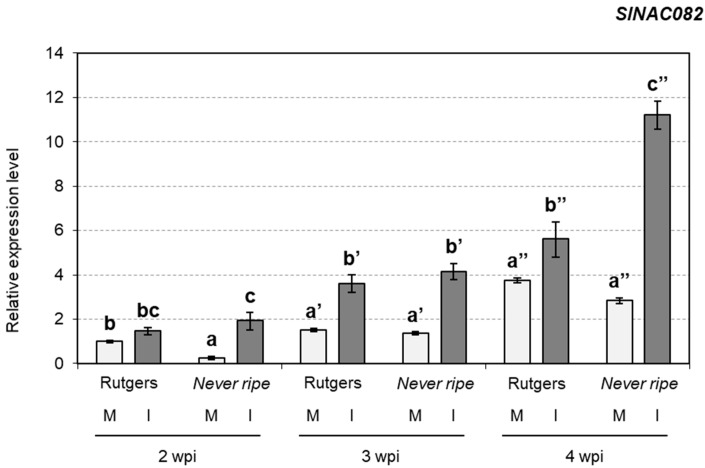
mRNA expression of *SlNAC082* in Rutgers and *Nr* plants from mock (M) and CEVd infected (I) plants determined by qRT-PCR at two, three and four weeks post inoculation (wpi). Expression levels are relative to Rutgers mock plants and normalized to the tomato actin gene. Data correspond to the mean of at least two independent plants ± SD of at least three technical replicates. Results corresponding to one representative experiment are shown. Data were analyzed using a Mann–Whitney test and different letters indicate significant differences (*p* < 0.05). Number of (**’**) indicates different weeks.

We previously reported that CEVd produces ribosomal stress in tomato plants [30]. To explore the possible role of ethylene in the ribosomal stress caused by viroids, *SlNAC082* (accession Solyc11g005920.1.1) expression levels were analyzed in both Rutgers and *Nr* mutant plants upon CEVd infection (Figure 8). The expression levels of *SlNAC082* were higher in CEVd infected plants in both genotypes at any time point, especially in infected *Nr* mutants when compared to infected Rutgers plants at 4 wpi.

**Figure 9:**
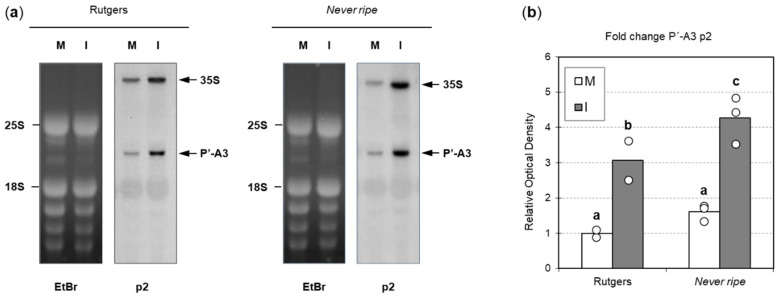
Alterations in tomato rRNA processing upon CEVd infection. (**a**) RNAs from Rutgers (left) and *Nr* (right), mock (M) and CEVd infected (I) tomato leaves were separated on an agarose gel, stained with ethidium bromide (EtBr) for the visualization of the 25S and 18S mature RNAs (left panels), and then were hybridized with the p2 probe for the detection of pre-rRNA 35S and P’-A3, marked on the right with an arrow (right panels). (**b**) Quantification of P’-A3 accumulation in Rutgers and *Nr* plants. Data correspond to the mean ± SD of 2–3 biological replicates and their individual values (open dots). Results were analyzed using a Mann–Whitney test and different letters indicate significant differences (*p* < 0.05).

To investigate whether rRNA processing is further affected in *Nr* mutants, a Northern blot analysis was performed with a probe targeting the P’-A3 pre-rRNA (see Materials and Methods) (Figure 9a). As previously described [30], an overaccumulation of 35S pre-rRNA and P’-A3 was observed in CEVd-infected plants. Results were quantified by optical density analysis and a statistically higher accumulation of P’-A3 was observed in *Nr* mutants compared to Rutgers plants (Figure 9b). These results suggest a defect in ribosome processing during CEVd infection, with a greater impact in *Nr* mutant plants.

In conclusion, we have observed that *Nr* mutants display hyper-susceptibility to CEVd infection, displaying a higher induction of *PR1*, an increase in the activation of the ethylene biosynthetic pathway, and enhanced ribosomal stress, thus indicating that ethylene plays a key role in mediating disease development upon CEVd infection in tomato plants.

## 3. Discussion

The involvement of ethylene in viroid disease has already been described for CEVd [15] and other viroids [22,44], although its precise role has not been deciphered yet. Previous studies have shown the capacity of ethylene to provoke similar symptoms to those caused by viroid infection [9,45]. On the other hand, recent studies have revealed some correlation between CEVd presence, ribosomal stress and symptom development [30]. However, the relationship between ribosomal stress and ethylene production had not yet been clarified. Hence, the goal of this study was to explore the role of ethylene in disease development and ribosomal stress upon CEVd infection.

The ethylene insensitive *Never ripe* tomato mutants, which constitutively block ethylene response, were used to investigate the role of this phytohormone in symptom development. The importance of ethylene in the plant defensive response to different pathogens has been studied using these mutants, which showed less susceptibility to diseases caused by bacteria [24,46] but higher susceptibility to symptoms caused by fungi [7,23]. Our results suggest that *Never ripe* mutants are more susceptible to CEVd infection than their corresponding wild type plants (Figure 1), supported by the accelerated appearance of symptoms (Figure 2), the greater severity of developed symptoms (Figure 3), and the higher expression and accumulation of PR1 (Figure 5). This susceptibility seems to be specific to CEVd, since *Never ripe* plants infected with TCDVd have been reported to display slightly reduced symptoms [22], thus indicating differences in pathogenicity between members of the *Pospiviroidae* family. Because ethylene perception is impaired in these mutants, our results suggest a role of ethylene signaling in the defensive response against CEVd.

Contrary to the hyper-susceptibility observed in *Never ripe* mutants, CEVd accumulation was higher in infected parental plants (Figure 4), suggesting that the symptomatology may not be associated with pathogen levels, as described by other authors [5,47]. This could be explained by a faster weakening of infected *Never ripe* plants, which displayed enhanced ethylene-related symptoms, therefore affecting the replication capabilities of CEVd.

The analysis of ethylene production showed higher ethylene levels in CEVd infected *Nr* mutants when compared to wild type plants (Figure 7), which correlates with higher *ACS2* expression observed in these mutants (Figure 6). This indicates an over-activation of the ET biosynthetic pathway upon CEVd infection in mutant plants. Moreover, negative feedback regulation has been described for the biosynthesis of ethylene through the regulation of *ACS* expression [11,24]. Higher *ACS2* and ethylene levels in *Never ripe* mutants could be explained by an impairment in this feedback regulation, due to the lack of signaling. ACS2 is a key enzyme in the ethylene biosynthetic pathway that is specifically induced upon necrotrophic pathogen attack [13,48,49,50,51,52,53]. Regarding viroids, an induction in ethylene-related genes during PSTVd infection has also been described [44], which correlates with our observed results upon CEVd infection. In NahG tomato plants, which are unable to accumulate salicylic acid (SA), a dramatic increment in ethylene synthesis also occurred after CEVd inoculation [5], which could be explained by the described antagonism between the ET/JA and SA signaling pathways [7,46,54]. In accordance with our results, these NahG tomato plants displayed a positive correlation between ethylene levels and symptom development upon CEVd infection [5], which has also been reported in other plant–pathogen interactions [8]. Besides, the viroidal symptoms can be mimicked by exogenous treatments with the ethylene-releasing agent ethephon, thus suggesting a role of ethylene in disease development [9,45]. It has also been described that, although high ethylene levels contribute to symptom development, low levels of this hormone can prevent viroid infection [55]. Our results indicate that symptom development upon CEVd infection might be caused by ethylene accumulation, even when ethylene signaling is not occurring. Here, we propose that ethylene plays a dual role in defensive response, in which its signaling is necessary for the plant defense against the viroid, and its accumulation is associated with symptom development.

This dual role has also been proposed for salicylic acid during pathogen attack. According to that model, NPR1 would act as a key regulator in SA signaling and would be degraded by NPR4 when no SA is present and by NPR3 under high SA levels [56,57]. Similarly, ethylene concentration might elicit a different response severity to the pathogen. In fact, a structural analysis of ethylene response 1 (ETR1) in *Arabidopsis* revealed the possibility of several ethylene binding sites for each ethylene receptor dimer [58]. We suggest a dual role of ethylene, by which low ethylene levels could contribute to slowing disease progression, but high levels might exert a toxic effect to the plant even when ethylene signaling is impaired, thus indicating an alternate route for symptom development. This is supported by reports in which *Never ripe* mutants from different cultivars retain slight ethylene sensitivity only under high ethylene concentrations [23,59,60]. Ethylene receptors work in homodimers [61,62] and have also been shown to form heterodimers [63,64,65]. ETR1 structural analysis showed that each monomer in the dimer may bind ethylene separately. It also reported an extra binding site between both monomers in the event of high ethylene concentrations [58]. Even though the main binding site of the NR receptor is impaired in *Nr* mutants, ethylene binding to the other monomer in heterodimers could explain the conserved sensitivity. At the same time, ethylene binding between monomers occurring under high ethylene concentrations may activate an alternate signaling route and indicate the possibility of ethylene binding to putative binding sites under high concentrations.

Ribosomal stress upon CEVd infection had already been described in previous studies [30]. However, no information on the relationship between this stress and ethylene had yet been reported. Here, we have detected that ET-insensitive *Never ripe* tomato mutants displayed higher levels of the ribosomal stress marker gene *SlNAC082* [31] once the disease was sufficiently advanced (Figure 8), although no differences were observed between *Nr* and wild type plants for earlier infection times. These results appear to indicate that differences in *SlNAC082* expression between *Nr* and wild type infected plants mainly occur at the late stages of infection, when a strong symptomatology is established. *ANAC082* is also involved in senescence in *Arabidopsis* [66], explaining the increase over time of *SlNAC082* expression levels in non-infected plants. Besides, *SlNAC082* induction correlates as well with higher *ACS2* expression and higher ethylene levels, suggesting a role of ethylene in ribosomal stress.

We have also studied whether ribosomal stress was due to defects in rRNA processing. In ribosome biogenesis, three of the rRNAs (18S, 5.8S and 25S) are processed from a single primary transcript consisting of a 5′-external transcribed spacer (5′-ETS), the 18S sequence, an internal transcribed spacer (ITS1), the 5.8S sequence, ITS2, the 25S sequence and a 3′-ETS [31,33]. Previous research showed a defect in 18S processing upon CEVd infection [30], which led us to analyze the accumulation of the immature pre-rRNA 35S and the intermediate P’-A3 (Figure 9). As expected, a higher accumulation of both 35S and P’-A3 was observed in CEVd-infected plants when compared to non-infected plants. Moreover, *Never ripe* mutants also displayed a higher accumulation of both intermediates, correlating with the higher ribosomal stress observed at the same time point (Figure 8). Our results confirm that CEVd symptomatology correlates with a defect in the processing of the 18S rRNA. This effect is higher in ethylene-insensitive mutants than in wild type plants, despite their lower CEVd accumulation. Our results suggest that ethylene signaling might be necessary to alleviate the ribosomal stress caused by CEVd. On the other hand, the correlation between higher *SlNAC082* expression and higher ethylene levels could also point to ethylene accumulation contributing to ribosomal stress, due to the dual role of ethylene previously proposed.

In conclusion, our results reveal the relevance of ET against the infection caused by CEVd, since ethylene insensitive *Never ripe* tomato mutants, which overproduce ethylene, display more severe symptoms compared to their wild type. This is not common to all viroids, indicating specific pathogenicity in CEVd. Besides, *Nr* plants also exhibit enhanced ribosomal stress, due to alterations in the 18S rRNA processing caused by CEVd despite the lower accumulation of viroid transcript, suggesting additional causes for the defects in pre-rRNA processing observed, and indicating an implication of ethylene in the defense against this stress caused by CEVd. We propose a dual role of ethylene in defensive response, by which low ethylene levels could delay plant disease development and mitigate the ribosomal stress caused by CEVd, while high ethylene levels may contribute to symptom severity and ribosomal stress.

## 4. Materials and Methods

### 4.1. Plant Material and Viroid Inoculation

Seeds of tomato (*Solanum lycopersicum* L.) cultivar Rutgers and the mutant *Never ripe (Nr)* were obtained from the Tomato Genetics Resource Center, UC Davis (https://tgrc.ucdavis.edu; accessions LA3001 and LA1090, respectively). Seeds were sterilized with a 1:1 mixture of commercial sodium hypochlorite and distilled H_2_O, and plants were grown in pots with a mixture of vermiculite and peat (1:1), which were irrigated with Hoagland solution.

A total of 35 Rutgers and *Never ripe* plants were used for each experiment. Plants were cultivated in a growth chamber with a 16 h light and 8 h darkness photoperiod and a temperature and relative humidity range of 28 °C/24 °C and 60%/85% (day/night), respectively. Viroidal inoculum was prepared from leaves of CEVd infected Rutgers tomato plants as previously described [67]. Ten plants were mock-inoculated with water and the rest were infected with CEVd (accession S67446) by inoculating with *carborundum* the first cotyledon and the first leaf of 2-week-old plants [5]. The apex and the two youngest leaves were sampled for all measurements throughout the experiment. Plants were inspected and symptom severity was scored at 1.5, 2, 2.5, 3 and 3.5 weeks post-inoculation (wpi) using the following scale: no symptoms (0), mild epinasty (1), severe epinasty and stunting (2), leaf rugosity (3), midvein necrosis and chlorosis (4).

### 4.2. Ethylene Measurements

Rutgers and *Nr* leaflets (0.5 g) showing symptoms of CEVd infection were harvested as previously described [5]. Samples were placed in the growth chamber for 4 h inside 10-mL glass vials sealed with a rubber septum, and 400 μL of the gas phase was analyzed. A 4890A Hewlett Packard gas chromatograph fitted with a flame ionization detector (FID) with a Teknokroma capillary column (2 m × 1/6” OD × 1 mm ID, Alumina F1 80/100) was used for ethylene measurements. Helium was used as the carrier gas with a pressure of 140 kPa. Injector and detector temperature were set at 200 °C and oven temperature was set at 80 °C. The ethylene peak retention time under these conditions was 2.5 min. Three replicates were measured for each time point and recorded data were analyzed with the Masslynx Waters software, using an ethylene standard curve.

### 4.3. RNA Preparation

Total leaf RNA was extracted using TRIzol reagent (Invitrogen, Carlsbad, CA, USA) and following the manufacturer’s protocol. RNA used for RT-PCR and qRT-PCR analysis was precipitated using one volume of 6 M LiCl and incubated for 3 h at 4 °C. RNA was recovered by centrifugation for 10 min at 12000 rpm and cleaned with 3 M LiCl. RNA was dissolved in DEPC water and measured using a ND-1000 Nanodrop. Concentration was adjusted to 1 μg/μL and DNA contamination was eliminated using the TURBO DNAse kit (Ambion, Austin, TX, USA), according to the manufacturer’s protocol.

### 4.4. RT-PCR and qRT-PCR

cDNA was synthesized from 1 μg of the extracted RNA using the *PrimeScript* RT kit (PerfectReal Time, Takara Bio Inc., Otsu, Shiga, Japan) following the manufacturer’s protocol, using the (dT)_18_ and random primers. A volume of 25 μL was used for RT-PCR using 1 μL cDNA, 1 μL of each primer, 3 μL dNTPs 2.5 mM, 0.5 μL DNa polymerase and 2.5 μL of its reaction buffer 10X (Netzyme, NEED, Valencia, Spain). Reactions were carried out using a GeneAmp PCR System 2400 (Perkin Elmer, Norwalk, CT, USA) thermocycler, using the following conditions: 1 min at 94 °C followed by 30 cycles of 1 min at 94 °C, 1 min at 56.35 °C, 1 min at 72 °C and a final extension step for 5 min at 72 °C.

Quantitative qRT-PCR was carried out as previously described [68] in a 10 μL volume, using MicroAmpFast 96-Well ReactionPlate (Applied Biosystems, Foster City, CA, USA) plates and PyroTaq EvaGreen qPCR Master Mix (CMB, Madrid, Spain) in a 7500 Fast (Life Technologies, Singapore, Singapore). Actin was used as the endogenous gene of reference. Primers used are shown in Table 1.

### 4.5. Northern Blot Hybridization

To detect P’-A3 in total RNAs preparations, 15 μg of RNA extracted from 4 wpi plants were denatured at 65 °C for 15 min, using 4 volumes of sample buffer (50% formamide, 6% formaldehyde in 200 mM MOPS, 50 mM sodium acetate and 10 mM EDTA, pH 7.0) and separated in a 1.5% agarose gel. Equal sample loading was checked by ethydium bromide staining and UV visualization. Transferring of RNA to Nytran membranes and hybridization was performed as previously described [69]. The p2 probe (5′-GAGCGCGGCAGTCATTCGCAAGGAGCATTC-3′) was labelled by using polynucleotide kinase and [γ-^32^P]-ATP. Membranes were exposed to X-ray film and optical density corresponding to three independent repetitions was analyzed by using the ImageJ software.

### 4.6. Protein Extraction and Electrophoresis Analysis

Proteins were extracted from tomato leaf tissues infected with CEVd and mock-inoculated plants. To this purpose, 0.3 g leaf tissue was homogenized in 1 mL of 50 mM Tris-Hcl (pH 7.5) containing 15 mM 2-mercaptoethanol, transferred to 1.5 mL tubes and centrifuged at 12000 rpm and 4 °C for 10 min. After this, 500 μL supernatant were transferred to a new 1.5 mL tube, then 250 μL of 3X SDS/PAGE loading buffer were added and the mixture was boiled at 95 °C for 7 min. Thirty-five μL of each sample, along with a molecular weight marker (PageRuler, Fermentas, Burlington, ON, Canada), were run in a 14% polyacrylamide gel, as previously described [70]. Gels were stained with Coomassie Brilliant Blue R-250 (Sigma-Aldrich, Chesnes, France), prepared at 0.05% in 10% acetic acid and 20% isopropanol.

### 4.7. Statistical Analysis

IBM SPSS Statistics 25 software was used for all statistical analysis. A *p* value < 0.05 was considered as statistically significant. The Shapiro–Wilk test was used for sample normality. The Mann–Whitney test was used to compare two independent non-parametric samples. A multiple group non-parametric comparison was performed by using a Kruskal–Wallis test.

## Figures and Tables

**Table 1:** Primer sequences used for real-time quantitative PCR.

Gene	Forward Primer (5′-3′)	Reverse Primer (5′-3′)
*Actin*	CTAGGGTGGGTTCGCAGGAGATGATGC	GTCTTTTTGACCCATACCCACCATCACAC
*PR1*	ACTCAAGTAGTCTGGCGCAACTCA	AGTAAGGACGTTGTCCGATCGAGT
*ACS2*	GATGGATTTGCGTCCACTTT	GATCCAGGCGAGACGTTAAG
*ACO1*	TGTCCTAAGCCCGATTTGAT	TTGAGGAGTTGAAGGCCACT
*CEVd*	AGGAGCTCGTCTCCTTCCTT	CACCGGGTAGTAGCCAGAAG
*SlNAC082*	TGCTGAAACCATTGGAACTG	CCAAGGAATTGCTTCCAAAA
